# Hide-and-seek: Threshold values and contribution towards better understanding of recovery rate in microplastic research

**DOI:** 10.1016/j.mex.2021.101603

**Published:** 2021-12-11

**Authors:** Inta Dimante-Deimantovica, Natalija Suhareva, Marta Barone, Ieva Putna-Nimane, Juris Aigars

**Affiliations:** a Latvian Institute of Aquatic Ecology, Agency of Daugavpils University, 4 Voleru Str., Riga LV-1007, Latvia; b Institute of Biology, University of Latvia, 1 Jelgavas Str., Riga LV-1004, Latvia; c The Faculty of Natural Sciences and Mathematics, Daugavpils University, 1 Parades Str., Daugavpils LV-5401, Latvia

**Keywords:** Microplastics, Environmental samples, Filtration, Density separation

## Abstract

Microplastic pollution has become one of the most pressing environmental issues. A fundamental criterion for risk assessment is the concentration of found microplastic that can be altered during microplastic isolating from the sample. Recovery rate (i.e. positive control) is an important feedback component that identifies accuracy, quality and efficiency of sample processing, same as physical and chemical impact. Here, using 100 µm red polystyrene (PS) beads we have tested some methodological steps that can be responsible for the possible microplastic losses during sample treatment and based on that, we provided a recovery rate threshold values. Our results support that the choice of the extraction method (vacuum filtration versus wet sieving) results in lower recoverability when vacuum filtration is used and that used separatory funnels size versus material amount impacts the efficiency or recoverability in density separation. We have also analysed microplastic recovery rate when different samples treatment steps from widely used isolation protocols (sediment and water) were applied and our results suggest that there are a number of factors affecting recovery rates, of which physical effects (loss by consecutive treatment steps due to material transfer) are more important than possible chemical degradation.

• Sample filtration method determines recovery rate from < 40 to > 80%.
• The number of sample processing steps involving transfer has a direct impact on recovery rate.
• As a measure of quality assurance, recovery rate thresholds are introduced.

## Graphical abstract

**Figure:**
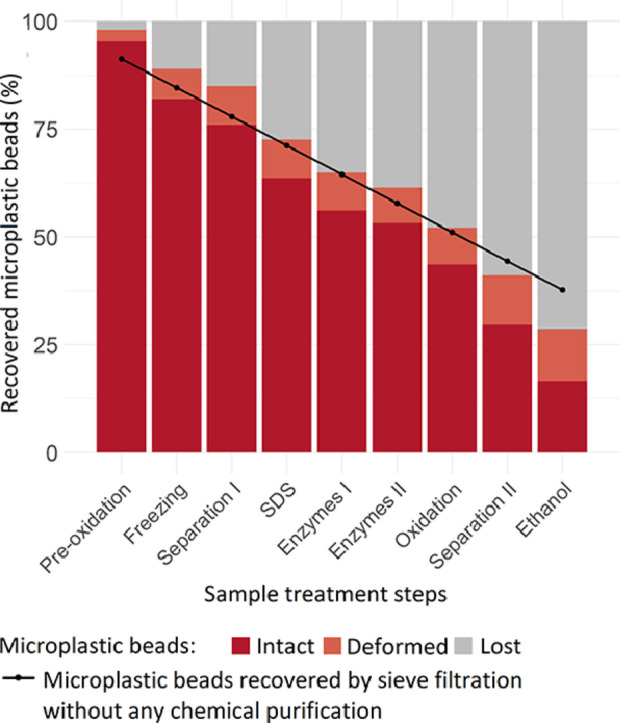

Specifications Table

**Table:** 

Subject Area:	Environmental Science
More specific subject area:	*Environmental pollution*
Method name:	*Recovery rate in microplastic research*
Name and reference of original method:	*Not applicable*
Resource availability:	*Not applicable*

## Method details

These days there are no doubts microplastics can move at different levels and can be found everywhere – atmosphere, water, soil, sediments, biota. In different environmental compartment samples microplastics must be separated from organic and inorganic matter in order to be quantified and characterized. Depending on the matrix and separation protocols employed it can be a long process with a number of sequential steps involved. Hence, it is important to evaluate accuracy and quality of this work to make results comparable and not misinterpret obtained data. There are several separation protocol steps that might affect the estimated number of microplastic particles in the sample, e.g. chemical digestion of the particles, mechanical degradation, loss during transfer, contamination from laboratory and operators [1,^5^,^8^,16]. This may consequently lead to unreliable under- or overestimation of microplastic pollution found in the environment. Therefore, it is crucial to evaluate efficiency of microplastic particles extraction across the studies.

As a measure of quality assurance, recovery rates (positive control) must be determined and reported. Numerous authors have pointed out the importance and necessity of spike-and-recovery approach as a part of standardized protocol for extraction of microplastic particles [2,^11^,^18^,25]. Still, the challenge remains since recovery success may differ due to protocol applied, variability of environmental matrices and polymer properties such as type, shape and size [3,25]. For instance, already Olesen et al. [20] pointed at recovery rate success decrease (96% for water protocol and 64% for sediment protocol) reflecting the impact of the number of total sample treatment steps. Wang et al. [29] concluded, that the efficiency is higher when extracting larger (100 µm) microplastic particles compared to smaller ones (0.05–4.8 µm). More effective recovery of a larger particles same as higher recoveries from sediments low in organic matter, but greater in grain size was reported by Cashmann et al. [3]. The same author also discussed issues as to fibre recovery when even larger particles (250–500 μm) are difficult to recover due to contrast of their diameter (20 μm). In general, there is a trend of increasing particles recovery with increasing solution density during density separation. However, larger particles of the same polymer (800–1000 μm) may also show decreased recovery rates compared to smaller particles (200–400 µm) depending on density separation solution used [24]. The impact of chemical degradation may also depend on particles size since smaller particles can be more affected by chemicals due to larger surface area versus volume. Wang et al. [29] found that only smaller microplastic particles experienced lower extraction efficiencies when oxidized with hydrogen peroxide (H_2_O_2_). Al-Azzawi et al. [1] selected particles of different polymers with sizes between 80 and 330 µm for potassium hydroxide (KOH) protocol treatment and observed, that the considerable part of polyethylene terephthalate (PET) and polylactic acid (PLA) were lost, e.g., presumably dissolved. When much larger (2850–3600 µm) PET particles were treated with KOH under comparable conditions done by Hurley et al. [8] no such effect was detected. Weber et al. [30] detected much more material is lost due to transfer and rinsing, when smaller particles are used (22–27 µm, recovery rate 53%) compared to larger particles (45–53 µm, recovery rate 89%).

Following early works done, the consensus is that without positive control, the essential criterion for quality assurance is lost. On the contrary, due to wide variety of affecting factors and results achieved, the spike-and-recovery approach cannot be directly applied to correspond to real samples. Still, consistency for recovery rates routine amongst studies would allow to compare and evaluate the quality of work. That would raise the concern that recovery rate results are not always reported [10,^18^,^26^,31] or they represent just part of the samples treatment protocol [12].

Our work primarily builds upon issues described above aiming to fill existing knowledge gaps for recovery rate as a measure of quality assurance in microplastic research. To do that, we tested several crucial obscurities in microplastic samples purification practice. First, we have tested most common treatment activities throughout different protocols, i.e. rinsing and filtering contribution to particles loss by comparing two sample extraction methods (in water matrix). Second, we analysed recovery rate success depending on material (sediment) amount versus separatory funnel volume since density separation is one of the most applied and sometimes the only method in the microplastic samples purification. Finally, in the water and sediment matrices we were considering the impact of reagents versus physical treatment to shed a light on treatment steps with greatest loss of particles and to test hypothesis that an increase in the number of treatment steps leads to a decrease in the overall recoverability. For that, prepared samples with added polystyrene beads (⌀ 100 µm) were processed in accordance with different protocols. In addition, we provide a threshold values for recovery rates results based on experiments we did. Our findings highlight not only the importance of recovery rate as a part of standardized microplastic samples treatment protocol, but also the need for systematic, commonly accepted approach to quality control performance and results interpretation.

## Materials and methods

### Chemicals, reagents and materials used

Hydrogen peroxide 30% (H_2_O_2_), stabilized (Carl Roth); heavy liquid – sodium polytungstate SPT-2 Na_6_[H_2_W_12_O_6_], granulate (TC-Tungsten Compounds); sodium dodecyl sulfate (SDS) solution 20% (CH_3_(CH_2_)_11_OSO_3_Na), pure (PanReac AppliChem); Tris(hydroxymethyl)aminomethane NH_2_C(CH_2_OH)_3_, pure (Firma Chempur); hydrochloric acid 37% (HCl), pure (Honeywell International Riedel-de Haën™); glacial acetic acid ≥ 99% (CH_3_COOH), pure (Firma Chempur); sodium acetate trihydrate (CH_3_COONa*3H_2_O), pure (Firma Chempur); iron (II) sulphate heptahydrate (FeSO_4__×_7H_2_O), pure (ES); sodium hydroxide (NaOH), pure (Firma Chempur); sulphuric acid 96% (H_2_SO_4_), pure (Carl Roth); hydrogen peroxide 50% (H_2_O_2_), stabilized (Scharlab); cellulase enzyme blend (Sigma-Aldrich), activity > 1000 U/ml; viscozyme L. cellulolytic enzyme mixture (Sigma-Aldrich), activity > 100 FBGU/g; alcalase enzyme *Bacillus licheniformis,* (Sigma-Aldrich Calbiochem®), activity > 0.75 Anson U/ml; chitinase enzyme, activity > 100 U/ml; Protease from *Bacillus* sp., (Sigma-Aldrich), activity > 16 U/g; ethanol absolute 99.8% (C_2_H_5_OH) (Sigma-Aldrich); glass fibre filters 1.2 µm, ⌀ 47 mm (Whatman®); stainless steel filers, mesh size 10 µm, ⌀ 47 mm (cut from stainless steel mesh sheets, Filtertek A/S, Denmark); woven wire mesh stainless steel sieve, mesh size 50 µm, ⌀ 100 mm (RETSCH production); red polystyrene (PS) beads ⌀ 100 µm density 1.05 g/cm^3^ (Sigma-Aldrich) – the particular size, shape and colour of the polymer was chosen due to well-defined shape and straightforward recognition.

### Quality assurance

In order to provide high quality assurance, all experiments were done according to the routine of specially prepared laboratory for processing microplastic samples. Airborne contamination is tested during experiments, samples are treated either in fume or laminar flow hoods (depending on the treatment step). Equipment used was made of glass, metal or polytetrafluoroethylene (PTFE) and was thoroughly rinsed with distilled filtered water prior to use. Samples were covered with aluminium foil at the time when not processed or when they were placed in a shaking heating bath. Cotton/linen laboratory coats of a specific colour (green and purple) and nitrile gloves were worn throughout the treatment activities by the laboratory personnel.

### Microplastic recoverability depending on extraction method (experiment 1)

According to most samples treatment protocols, the supernatant (sample) must be removed by filtration after each processing step to be processed further, which, in turn, can significantly affect the loss of particles during the transfer. Here we assessed effectiveness of two sample filtration methods: (a) by borosilicate vacuum filtration assembly (consisting of top funnel, filter holder, filtering flask) coupled with a stainless steel filter (10 µm mesh size, ⌀47 mm) and fixed by aluminium clamp (VWR production), (b) by wet sieving via woven wire mesh stainless steel sieve (50 µm mesh size, ⌀100 mm, RETSCH production). Prepared samples of 100 PS beads added to 100 mL of filtered Milli-Q water were used. The extraction procedure was repeated nine times for each method, simulating the freshwater sediment protocol no.1 (see further in the text in chapter Microplastic recoverability depending on different protocols applied (experiment 3)) with nine treatment steps. The whole experimental setup for each method was replicated three times.

**Fig. 1:**
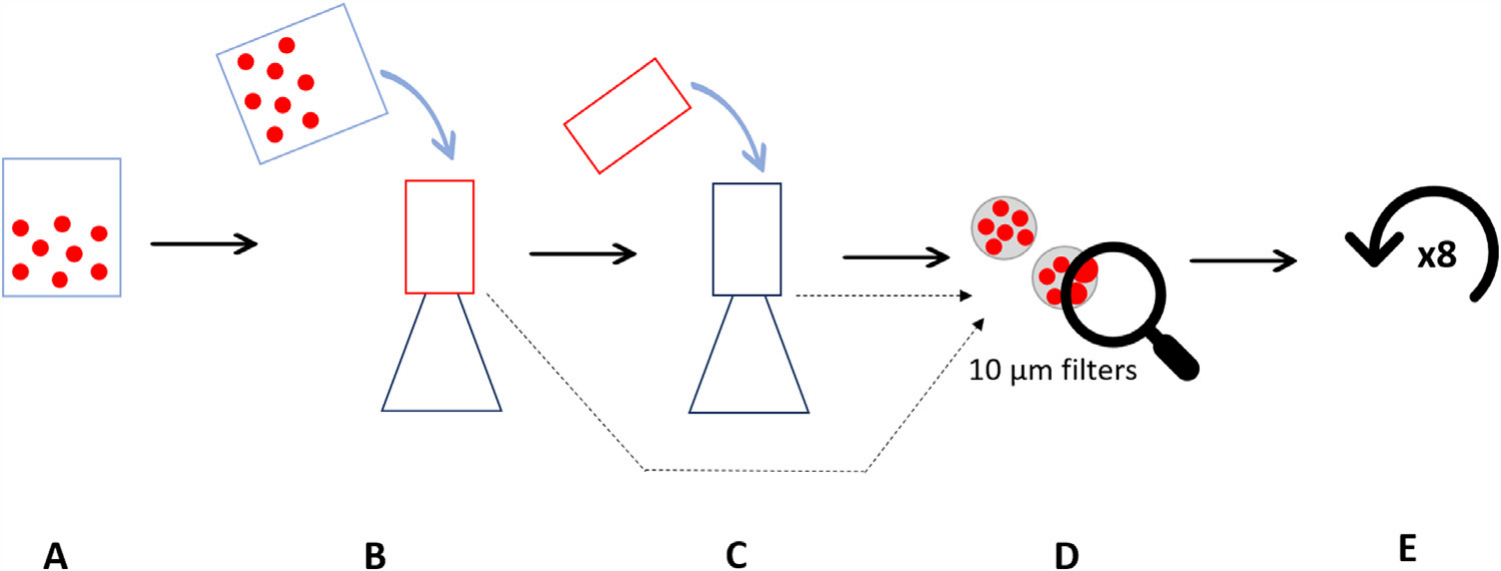
Experiment design for microplastic recoverability depending on extraction method - vacuum filtration. A – 100 polystyrene beads isolated in a beaker with 100 ml of filtered Milli-Q water; B – liquid containing beads filtered through 10 µm stainless steel filter; C – top funnel of the used filtering apparatus rinsed and filtered on another 10 µm stainless steel filter in a clean filtering apparatus; D – beads on filters counted under the microscope; E –process repeated.
(b) Sieve extraction – prepared sample was sieved through 50 µm mesh sieve, thoroughly multiple times rinsing the beaker to reduce the loss of beads as much as possible. Beads from the sieve were rinsed in a clean beaker and counted under a binocular. This process was repeated eight more times (Fig. 2).

(a) Vacuum filtration - sample was poured on a 10 µm filter in filtering apparatus, thoroughly multiple times rinsing the beaker and top funnel to reduce the loss of beads as much as possible. Filter was saved in a Petri dish. Top funnel of the used filtering apparatus was rinsed and filtered on another 10 µm filter placed in a clean filtering apparatus to make sure beads stuck in the connection points of the filtration assembly top funnel and filter holder are recovered as well. Both filters were further analysed under a microscope to register the number of beads found. After, the beads were flushed from the filters into a clean beaker to repeat the process eight more times (Fig. 1). The rinsed filters and Petri dish were checked additionally under a binocular to insure that no beads were left on the surface.

**Fig. 2:**
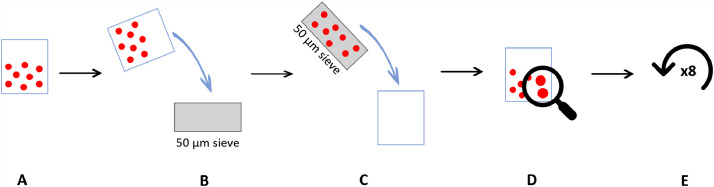
Experiment design for microplastic recoverability depending on extraction method – sieve filtration. A – 100 polystyrene beads isolated in a beaker with 100 ml of filtered Milli-Q water; B – liquid containing beads sieved through 50 µm stainless steel sieve; C –beads rinsed from the sieve in a clean beaker; D –beads counted using binocular; E – process repeated.

### Microplastic recoverability depending on sample volume versus separatory funnel volume (experiment 2)

**Fig. 3:**
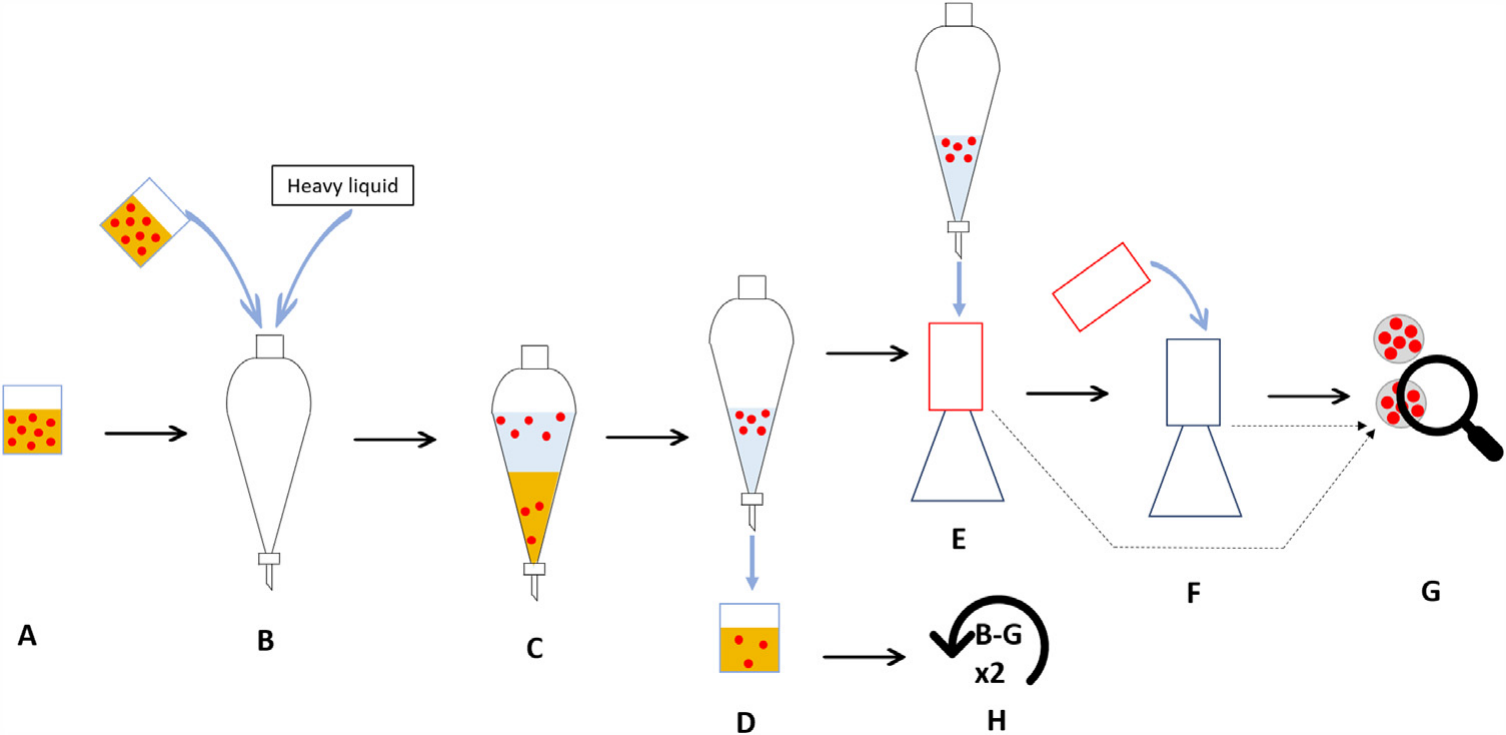
Experiment design for assessing microplastic recoverability for sample volume/weight versus separatory funnel volume. A – Sample material with 100 PS beads isolated in a beaker; B – material containing beads transferred to separatory funnel together with heavy liquid, aerated for 5 min; C – settling of sediments for 24 h; D – separation of settled sediments; E – filtering of supernatant containing PS beads on 10 µm stainless steel filter; F – top funnel of used filtering apparatus rinsed and content filtered on 10 µm stainless steel filter using another clean top funnel; G – counting beads on 10 µm metal filter under microscope; H - repeat steps B-G two more times with settled sediment sample (from D).

Density separation is amongst most applied methods in microplastic research and becomes more demanding when larger volume samples placed in larger separatory funnels are operated. For large value sample material and extensive treatment procedure it is crucial to take sub-samples of size representative enough. Simultaneously, it is important to keep protocol smoothly running at reasonable costs. This experimental design (Fig. 3) was developed to understand whether the volume of the separating funnel itself (with a constant wet sediment sample volume) is important. Wet natural (marine) sediment samples (50 ml) with an average dry weight of 8.65 ± 0.54 g were spiked with 100 PS beads each and through the glass funnel were placed from glass beaker in 250 mL, 500 mL and 1000 mL conical separatory funnels. Both glass funnels and glass beakers were thoroughly multiple times rinsed with the heavy liquid to make sure all material gets into the separatory funnels. Thereafter, separatory funnels were filled with a heavy liquid up to two thirds of the volume. The filled funnels were aerated by shaking manually for five minutes, then placed on a holder and left to settle for 24 h. The settled part of the sample was drained off through the bottom of the funnel. The supernatant containing beads was filtered (vacuum filtration assembly) through a 10 µm filter, thoroughly rinsing the inside of the funnel using filtered Milli-Q water. Top funnel of the used filtering apparatus was rinsed and filtered on another 10 µm filter placed in a clean filtering apparatus. The collected beads were counted to calculate recovery rate. The separation was performed three times for each sample using the same separated sediment part, placing it back in the separatory funnel, fixing heavy liquid amount added and performing aeration. The whole experimental setup was done twice (Fig. 3).

### Microplastic recoverability depending on different protocols applied (experiment 3)

Here we tested the hypothesis that recovery rate depends on protocol in terms of the number of consecutive treatment steps applied. This goes hand in hand with environmental compartments since there are matrices requiring less treatment steps (e.g. water) and much more treatment steps (e.g. soil and sediments). We wanted to see if physical treatment has more effect compared to chemical impact and at what treatment stage the greatest loss of particles will occur.

Each sample consisted of 100 mL filtered Milli-Q water containing 100 PS beads. Samples were processed following already existing and in daily routine used sample processing protocols (adapted from [7,^13^,^14^,^17^,23]): (a) freshwater sediment protocol no.1, (b) freshwater sediment protocol no. 2, (c) marine sediment protocol, (d) marine surface water protocol. Protocols involved different number of treatment steps and chemicals used. After each treatment step, the samples were filtered through a 10 μm filter by vacuum filtration assembly to remove previous supernatant. Recovered beads were counted under the microscope to register the decline of recoverability (intact and deformed beads were registered separately). Following, the beads were flushed from the filter in a clean beaker to continue the treatment process according to the protocol. When transferring sample, all beakers and top funnels used were rinsed thoroughly multiple times.

(a) Freshwater sediment protocol no.1 includes eight consecutive purification steps: pre-oxidation by H_2_O_2_, freeze-drying, density separation 1, SDS-treatment, enzymatic treatment in TRIS buffer, enzymatic treatment in acetate buffer, Fenton reaction, density separation 2 and one preservative step – fixation by ethanol. The whole experimental setup was represented by four replicates.
  First, 30% H_2_O_2_ (50 mL) was added to the samples. Second, the samples were incubated in a shaking water bath (for 24 h at +50 °C, 100 rpm). After that, the beads were flushed with filtered Milli-Q water into a clean beaker,frozen at −20 °C and placed in a vacuum freeze-dryer for 48 h.Next, samples were filtered through a 10 μm filter by vacuum filtration assembly and flushed from the filter into a separatory funnel with heavy liquid (density 1.75 kg L^−1^), reaching up to two-thirds of the funnel volume. The funnel was aerated by shaking manually for 5 min, then placed on the holder and left for 24 h to settle. After that, the settled part of sample was drained off and saved for further separation. The top layer was poured onto a vacuum filtration assembly with a 10 μm filter, thoroughly rinsing the inside of separatory funnel using filtered Milli-Q water. The settled part was placed back into the separatory funnel, aerated for 5 min and left to settle for another 24 h. Each sample was separated three times. After each density separation, beads from the 10 µm filters were flushed with 5% SDS solution (maximum total volume 200 mL) into a beaker; then the samples were incubated in a shaking water bath (for 48 h at +50 °C, 100 rpm). In the next step, the beads were flushed with 300 mL of TRIS buffer (pH 8.2) from the filter into a clean beaker, then 0.5 mL of alcalase was added; the samples were incubated in a shaking water bath (for 48 h at + 50 °C, 100 rpm). Next, the beads were flushed with 300 mL of acetate buffer (pH 4.8) from the filter into a clean beaker, then 0.5 mL of viscozyme and 0.5 mL of cellulase were added, the samples were incubated in a shaking water bath (for 48 h at + 50 °C, 100 rpm). After that, the beads were flushed with 200 mL of filtered Milli-Q water from the filter into a 1 L beaker, cooled to 15–20 °C and then 145 mL H_2_O_2_ (50%), 65 mL of 0.1 M NaOH and 62 mL of 0.1 M FeSO_4_ were added to the solution. The samples were kept within temperature range 20–30 °C for at least 4 h, then left to stand overnight. After filtration, the beads were flushed into a separatory funnel with heavy liquid (1.75 kg L^−1^) for a second separation cycle using the same procedure as was described above. Finally, the beads were washed with the filtered Milli-Q water and 50% ethanol solution and flushed from the filter into a clean beaker with 50% ethanol solution and left at 40 °C for two weeks.
(b) Freshwater sediment protocol no.2 is based on the freshwater sediment protocol no.1 with several slight modifications: the step 1 (pre-oxidation) and the step 2 (freezing) have been swapped, the density separation 1 and 2 consisted of two separations each (not three as in protocol no.1), during enzymatic treatment in TRIS buffer, both 0.5 mL of alcalase and 0.5 mL of protease was added, ethanol preservation was not included. The whole experimental setup was represented by three replicates.
(c) Marine sediment protocol is based on the freshwater sediment protocol no.1. Protocol includes five consecutive purification steps: density separation 1 (two separations), pre-oxidation by H_2_O_2_, enzymatic treatment in TRIS buffer (0.5 mL of protease only) enzymatic treatment in acetate buffer (0.5 mL of viscozyme, 0.5 mL of cellulase and 0.1 mL of chitinase), density separation 2 (one separation). The whole experimental setup was represented by three replicates.
(d) Marine surface water protocol consisted of three treatment steps – NaOH, H_2_O_2_ and enzymatic treatment in acetate buffer. The whole experimental setup was represented by three replicates.
  First, 10% NaOH (300 mL) was added to the samples. Second, the samples were incubated in a shaking water bath (for 24 h at + 50 °C, 100 rpm). After filtration, the beads were flushed with filtered Milli-Q water into a clean beaker, total volume of Milli-Q water was fixed to 100 ml and 30% H_2_O_2_ was added (200 ml). Sample was again incubated in a shaking water bath (for 24 h at + 50 °C, 100 rpm). For the final treatment step, 300 mL of acetate buffer (pH 4.8) and enzymes (0.5 mL of viscozyme and 0.5 mL of cellulase) were added to the filtered sample, followed by incubation in a shaking water bath (for 48 h at + 50 °C, 100 rpm).

Finally, to understand whether chemical treatment or the number of treatment steps has a stronger effect on recovery, the most intense protocol (freshwater sediment protocol no.1 with 9 treatment steps) was compared to a cycle of nine successive vacuum filtrations without adding any chemical reagent. For this purpose, along the percentage of recovered beads per each treatment step also the relative recoverability was calculated which is a percentage of beads that were lost compared to the previous treatment step.

### Statistical analysis and calculations

Mann Whitney U Test was used to compare the recoverability of sieving and vacuum filtration extraction methods and their regression curves for different number of treatment steps. The relationship tested was considered significant as the *p*-value was under 0.05. Linear regression analysis was performed to determine the mean recoverability pattern based on different processing protocols.

The threshold values for recovery (%) for standardized ⌀ 100 µm red PS beads were calculated from the regression curve of recoverability described above. The threshold values for sieve extraction method were further adjusted coupling with the regression equation obtained in experiment 1. The residual standard error (RSE) for the adjusted threshold values was assumed to be the same as that obtained from the regression curve of the recoverability calculated for the tested treatment protocols.

Data exploration, artworks, and statistical analyses were performed using R software for Windows, release 4.0.3.

## Method validation

### Microplastic recoverability depending on extraction method (experiment 1)

**Fig. 4:**
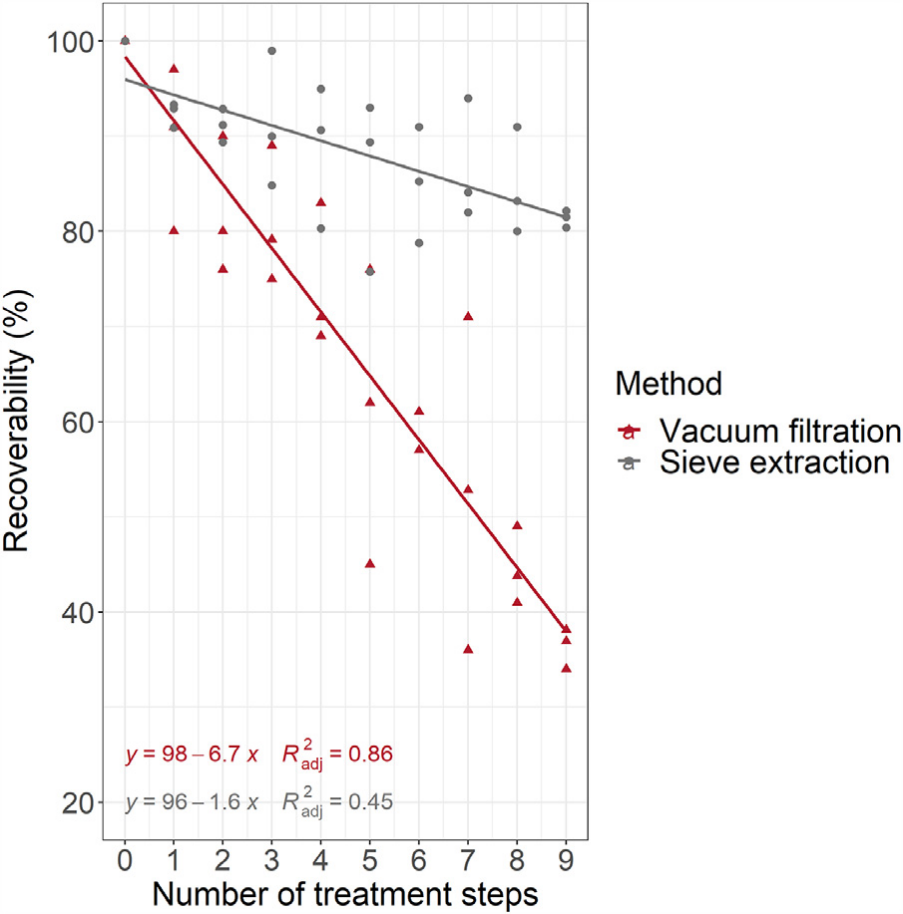
Recoverability (%) of the 100 polystyrene (PS) beads depending on extraction method (vacuum filtration and sieve extraction) during nine consecutive treatment steps.

In this experiment we compared two methods what can be used to filter and rinse samples during samples purification process, i.e. vacuum filtration and sieve extraction. As can be seen in Fig. 4 and according to Mann Whitney U Test, there is a significant difference (*W* = 172, *p*-value = < 0.001) between those two principal methods used. The vacuum filtration method exhibited a stronger negative slope correction (*y = 98 – 6.7‧x*. R^2^ = 0.86, *F* = 174.7, *p*-value < 0.001, RSE = 7.9) than the sieve extraction method (*y = 96 – 1.6‧x, R^2^ = 0.45, F = 24.9, p-value < 0.001, RSE = 5.1*), which resulted in lower recoverability of beads when vacuum filtration was used (Fig. 4). Thus, the difference of recoverability rate after first, fifth and ninth filtration between vacuum filtration and sieve is as follows: 91.3% and 94.4%, 64.5% and 88.0%, 37.7% and 81.6%. However, significant difference (*W* = 64, *p*-value = 0.045) in recoverability between both methods appears only starting with fourth filtration. Up to third filtration, there is no statistically significant difference (*W* = 47, *p*-value = 0.154). These results suggest that even without chemical treatment already considerable part of the material can be lost due to physical treatment (transfer) depending on filtration/rinsing method used. This is also supported by Weber et al. [30] in a study where vacuum filtration was used. Recovery rates for drinking water analysis (with a very minor chemical treatment) were established to quantify material loss through transfer and rinsing and resulted in 53% to 89% depending on particle size and counting approach [30]. Likewise, Thiele et al. [28] admitted the fall of the particles number could happen during accidental transfer. Another loss of particles may occur due to trapping in the mesh filters as suggested by Miller et al. [18], although we did pay attention to this and did not observe any trapped beads in the mesh filters. Trapping is also unlikely since standardized spherical shape beads of 100 µm were used (the mesh size of the filter was 10 µm while for the sieve it was 50 µm) in our study. Our findings indicate the recovery rate may differ greatly whether filtering system used is solid and close (sieve) or semi-close (vacuum filtration assembly consisting of compatible components). After vacuum filtration, top funnel of the filtering apparatus was always rinsed and filtered again on another filter placed in a clean filtering apparatus since we observed there are always some beads left in the connection points of the filtration assembly. Hence, we conclude, the semi-close filtering system increases accidental transfer risk and instance of losing particles. Our results support findings obtained by Nakajima et al., [19] who observed recovery rates higher for the sieve than the classical filter method both for the smaller (100–500 µm) and larger (500–1000 µm) particles. Although sieve effectiveness itself may be limited, e.g. Lusher et al. [15] showed when same mesh size sieves as stacking replicates are used, there are still particles found on all of them. It is also worth noting for closed filtering devices (sieve) the cleaning might be critical to ensure that following samples are not contaminated with previous material, hence sonication/high-temperature heating additionally to washing and rinsing would be advisable.

*Microplastic recoverability depending on sample volume* versus *separatory funnel volume (experiment 2)* In this experiment we wanted to see how the volume of the separating funnel itself affects the efficiency of the density separation.

**Fig. 5:**
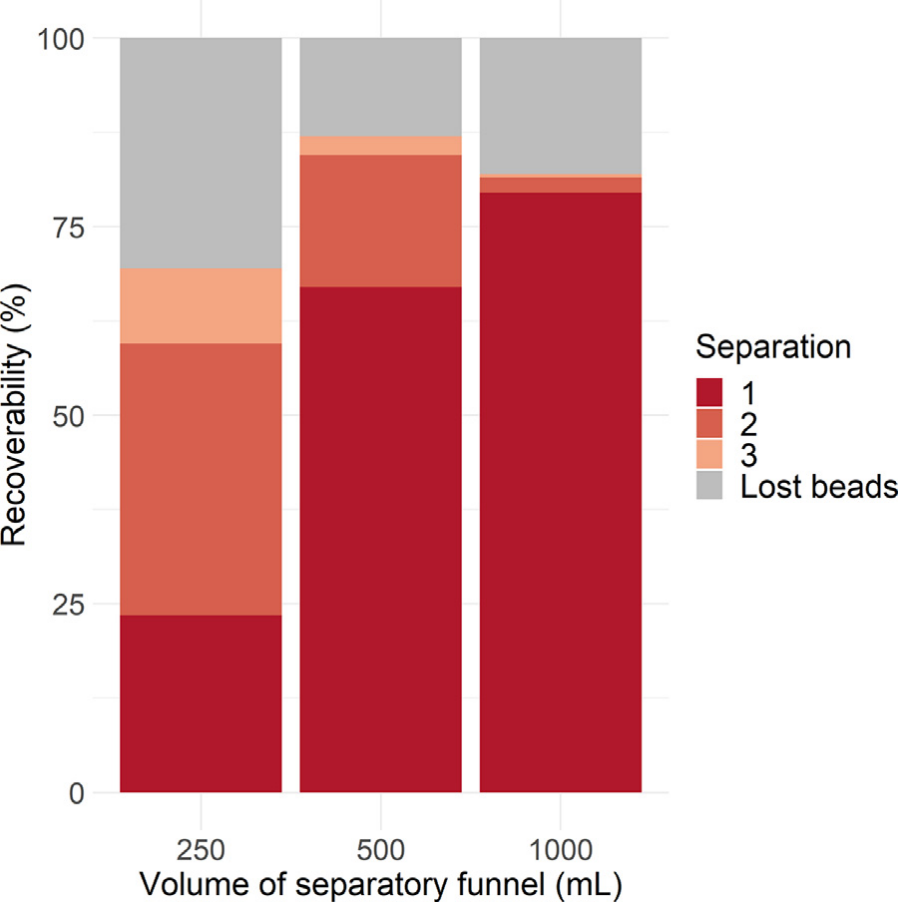
Recoverability (%) of the 100 polystyrene (PS) beads mixed in wet sediments after density separation in heavy liquid (1, 2, 3 – consecutive separation repetitions) using separatory funnels of different volumes.

Separatory funnels of volumes 500 mL and 1000 mL showed substantially higher total recoverability after three-step density separation with the constant sample size (on average 87 ± 6% and 82 ± 7%, respectively), compared to that of 250 mL funnel (on average 69 ± 19%) (Fig. 5). Moreover, the largest separatory funnel showed a high efficiency of the first separation step, when 97 ± 3% of the total amount of beads were returned. The first step efficiency for 500 mL funnel was equal to 77 ± 15% and for 250 mL funnel it was only 34 ± 0.4%. In our study case the separatory funnel volume was five (250 mL funnel) to 20 times (1000 mL funnel) the total volume of the liquids used. We assume the surface area in contact between the two liquid compartments (wet sediment sample and density separation fluid) increases much greatly in larger volume separatory funnel than in smaller one during aeration. Hence, recoverability already after first separation will be higher in large volume separatory funnel. However, this can be fixed by repeated separations (Fig. 5). In case of large volume samples, it is advisable repeating several separations using smaller portions of the sample. The results obtained clearly showed that the volume of the separatory funnel can affect recovery of microplastic particles and the overall efficiency of density separation. As mentioned earlier by Quinn et al. [24] it should be also noted that recovery success is influenced by the physical properties of sediments (granulometric composition) as well as microplastic particles (size, density, shape). In order to keep the density of heavy liquid more precise it is advisable to remove the water content of the sample by freeze drying before adding heavy liquid. Freeze-drying not only removes the water content from the sediments, but is also known to affect aggregate stability [4]. Enders et al. [6] mentioned that freeze-drying results in changed structure of the environmental material what in turn facilitates the following density separation performance. We could not observe possible fragmentation of microplastic beads due to freezing induced frost wedging (see in following chapter).

### Microplastic recoverability depending on different protocols applied (experiment 3)

**Fig. 6:**
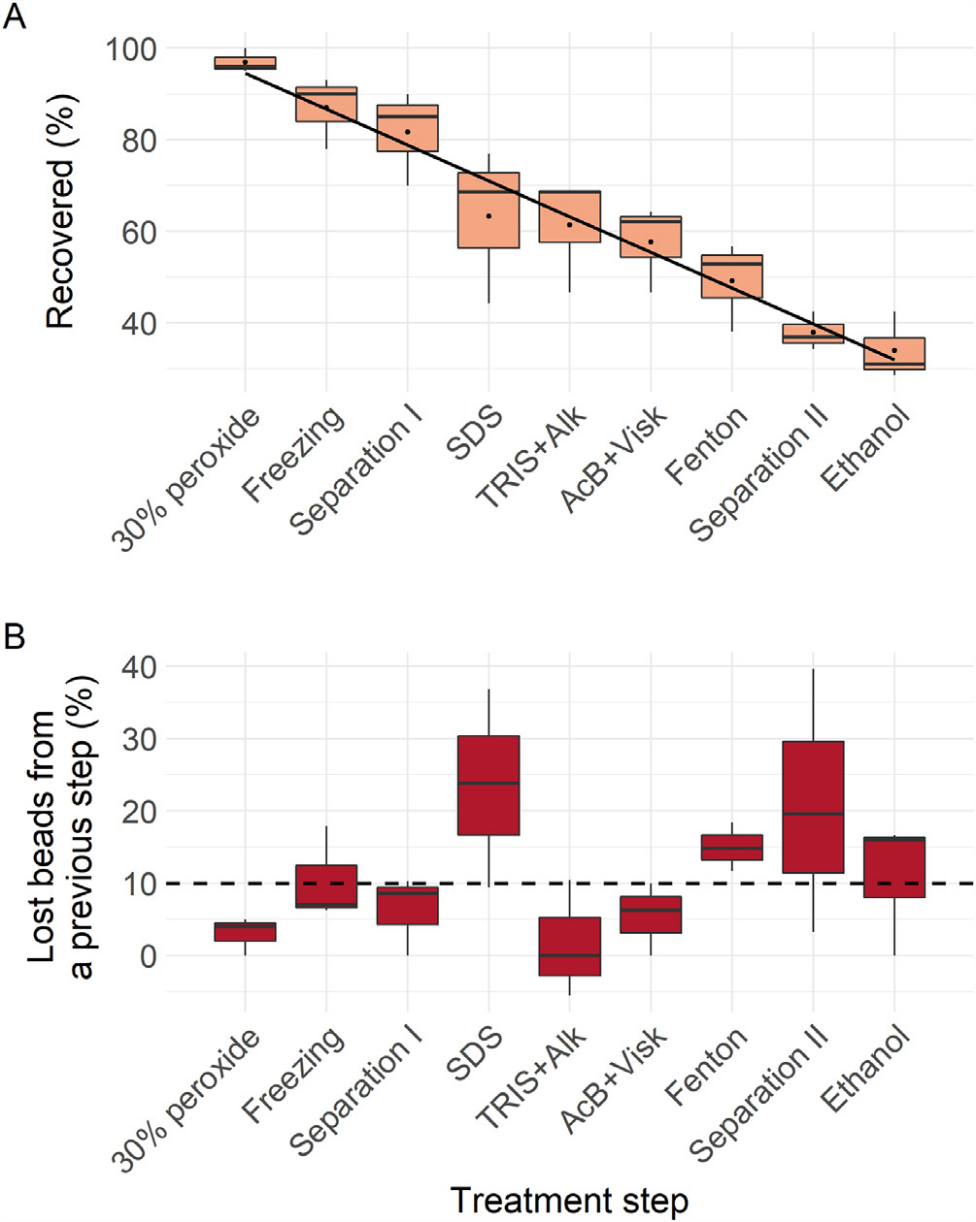
Percentage of recovered (intact) beads at different chemical treatment steps based on the results of freshwater sediment protocol no. 1 (*a*), where A – recoverability (%) compared to the initial number of beads; B – recoverability (%) compared to the previous treatment step; black dots and line – recoverability (%) at nine successive vacuum filtrations without adding any chemical reagent; black dashed line – more than 10% of the lost beads compared to the previous step were regarded as a substantial drop of recoverability.

Here, we tested which processing steps, if any, are most conducive to particle loss, and if the reduction in overall recoverability is directly protocol-dependant, i.e. depending on the number of treatment steps. Based on the results of freshwater sediment protocol no. 1 (*a*), recoverability showed a gradual decrease of intact beads (Fig. 6A and graphical abstract) without regard to treatment type. When data were compared to those of the first experiment (nine successive vacuum filtrations without adding any chemical reagent), recovery rate results turned to be coherent (see black line in Fig. 6A and graphical abstract).

**Fig. 7:**
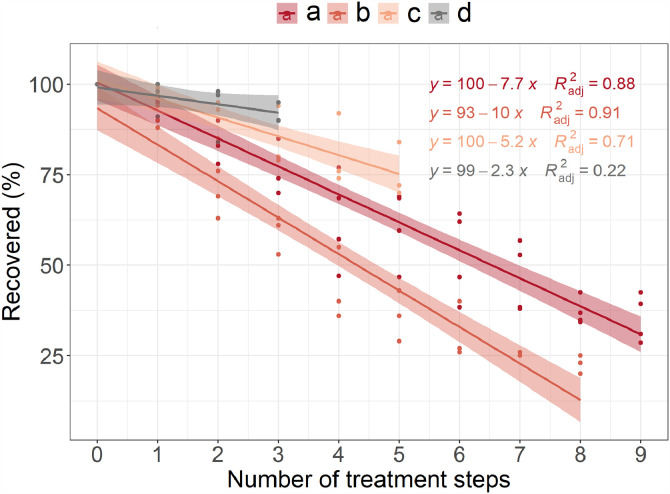
Regression curves of recoverability (%) depending on the number of treatment steps for different treatment protocols (two freshwater sediment protocols with nine and eight treatment steps (a, b), marine sediment protocol with five treatment steps (c) and marine surface water protocol with three treatment steps (d)).

Exploration of recoverability data showed a mutual decreasing pattern for all four treatment protocols used for different sample matrices (Fig. 7). Taken together, this is consistent with the hypothesis that an increase in the number of processing steps (physical impact) causes a reduction of the total recoverability or to be more precise – increase of material transfer number leads to recoverability decrease. It agrees with previous findings, e.g. Olesen et al. [20] showed that water protocol recoverability was 96%, while for fauna and sediment protocols it was 75% and 64%, respectively, thus, reflecting the impact of sample treatment steps/transfer number. Hence, we agree with Enders et al. [6], that sample treatment should involve as few steps as possible, especially if potential number of particles to be found is low. At least as long as treatment is associated with material transfer.

It is worth noting, that the impact of the chemical reagents, at least for protocols used in this study, played a minor role. Although, the relative recoverability indicated treatment steps when substantial drop (at least 10% and more compared to previous step) of the recovered beads was observed – SDS (sodium dodecyl sulphate), Fenton reaction, separation II and conservation in ethanol (Fig. 6B). SDS is a surfactant removing organic matter from the plastic particles and reducing adherence to lab equipment used. However, it is viscous and difficult to wash from lab equipment (beakers, filter compartments) itself, hence, increased loss of particles may occur during SDS step as increased number of particles adhere to walls of equipment used together with SDS. The Fenton reaction has been used widely and effectively to purify material removing biogenic organic matter. Its appropriateness and safety as to polymers degradation has been approved by previous studies [22,27]. Therefore, we assume, the last treatment steps in rather long protocol may result both in increased loss of the particles and number of deformed particles (see graphical abstract) due to cumulative effect. There are other possible important factors which may have a critical role, e.g. treatment time, temperature during the treatment and the concentration of applied chemical reagents [21]. The variation of those factors at different degree may definitely change the results of recoverability. Specially the increase of chemicals’ concentrations and temperatures results in accelerated degradation of plastic polymers as shown by Pfeiffer and Fischer [21]. Karami et al. [9] found that 10% potassium hydroxide (KOH) is efficient in digesting sample material at different temperatures, however, reaching 50 and 60 °C, it degrades polymers or reduces its recovery rate. In our experiment we have used currently common protocols confirmed to be safe for most of plastic polymers [7,^13^,^14^,^17^,23], therefore we exclude possible significant decrease of recoverability due to factors mentioned above.

Summarizing the entire data set of all protocols used (Fig. 7), we obtained the recoverability regression equation as follows:

$Recoverability\;\left(\%\right)=\;101.69-8.74\cdot N_{processing\;steps}\;\left(R^{2}=0.78,\;F=\;349.5,\;p<0.001,\;RSE=12.3\right)$, where, Recoverability (%) – estimated recoverability of method, $N_{processing\;steps}$ is the number of processing stages in the selected method. Estimated levels of recoverability (%) thresholds based on the number of chemical treatment steps are presented in Table 1.

**Table 1:** Recovery rate threshold values (%) for standardized red polystyrene (PS) beads ⌀ 100 µm, based on the total number of processing stages in a selected method (semi-closed (vacuum assembly) or closed (sieve) filtration method) ^1^ RSE – Residual Standard Error.

Number of processing stages	Recoverability (%), semi-closed (vacuum) filtration, RSE = 12.3	Recoverability (%), closed (sieve) filtration,estimated RSE = 12.3
1–3	93–75 ± RSE	
1–6		96–78 ± RSE
4–6	67–49 ± RSE	
7–9	41–23 ± RSE	74–67 ± RSE

We divided the number of treatment steps in three groups (1–3, 4–6, 7–9) due to the reason there was a significant difference in recoverability between sample extraction methods starting with treatment activity number four.

However, some limitations are worth noting. This is persistent if the rest of the workflow (density separation, chemical digestion modules, e.g. acid, oxidative, alkaline and enzymatic) is approved to be safe and not harmful to microplastic particles. Also widely accepted quality assurance and quality control measures have to be considered [2]. Particles used in our experiments were of single size range (100 µm), shape (beads) and polymer type (PS), hence, most likely, the results would vary when different size, shape (particularly fibres) and polymer type (density differences) groups would be involved. Depending on the orientation, fibres, for instance, can easily pass even small size mesh and therefore can result in low recovery compared to other shape plastic particles as in the study by Lares et al. [11]. In the same study authors are suggesting the possible solution – the use of cascade sampler with decreased mesh size. Besides, our peripheral observations suggest, the operators experience matters as well for higher recovery rates.

## Conclusions and recommendation for future work

In this study we wanted to contribute to the existing knowledge on recovery rate as positive control in microplastic research quality assurance. We have discovered several procedures in samples treatment protocols that significantly impact the results of recoverability (sample extraction methods, sample volume versus separatory funnel volume in density separation, dependence of the overall recoverability on the total number of processing stages in a selected method). Based on results obtained, we have provided recovery rate threshold values that can be directly applied under the same conditions as in the present study.

It is clear (and ordinary), that with the current sample purification methods widely used in microplastic research, we might lose part of our initial material and results might vary a lot. Samples treatment involves many steps and therefore – many material transfers, while environmental matrices may vary (e.g. the content and amount of organic matter). Therefore, it is recommended before samples treatment to test matrix in order to decide on optimal protocol (treatment steps/transfers involved) – a compromise between well enough (for further spectral analysis) purified sample and as little as possible lost material. The, results obtained across studies are rather trends than absolute number and difficult to compare. The application of recovery rates principle as positive control should be standardized.Although, artificially introduced particles for recoverability tests may give different results compared to naturally occurring particles in the environmental compartment samples. Therefore, recovery rate results may rather reflect the quality and appropriateness of a particular laboratory and protocol used than aids in material loss comparison across studies. Hence for future research we suggest to establish and introduce standardized positive control test kits consisting of few most common polymers of different shape (e.g. beads, fragments and fibres) and different size classes depending on research to be conducted (i.e. - range of target particles size). These kits can be used as standard additions to samples giving recovery rate for each specific sample. There are no doubts there will be still a lot of uncertainties of quantification since microplastic particles found in different matrices will vary a lot even from standardized test kits due to differences in polymer types, size, shape, fragility, weathering effect etc. Not least, results of recovery rates and detailed protocol description (including number of material transfer mentioned) should be reported alongside data obtained. To interpret recovery rates results even from adapted standardized control test kits when appropriate/particles safe sample treatment methods are used, threshold values must be introduced depending on samples treatment steps number and filtration approach. Threshold values (first to our knowledge) presented here, can be used as a starting point for further development towards better understanding of quality assurance and control.

## Declaration of Competing Interest

The authors declare that they have no known competing financial interests or personal relationships that could have appeared to influence the work reported in this paper.
